# Bebop on the Hockey Pitch: Cross-Disciplinary Creativity and Skills Transfer

**DOI:** 10.3389/fpsyg.2016.00123

**Published:** 2016-02-09

**Authors:** Clive M. Harrison

**Affiliations:** Department of Design, Communication and IT, University of Newcastle, NewcastleNSW, Australia

**Keywords:** creativity, transfer, domain-general, jazz, hockey

## Abstract

This paper generalizes task-specific (but dissimilar) skills, from the jazz concert stage and from the hockey field, into the domain of creativity research. What is sought are clues to *what skills or creativities are transferable across dissimilar domains*. It is argued that certain domain-general skills are transferable across domains, but a domain-general or ‘c’ creative capacity, is not. Rather than transferring some over-arching capacity to be universally creative, this research highlights factors likely to facilitate successful cross-disciplinary creative expression and posits a correlation between the capacities for discriminant pattern-recognition, task-specific expertise, and sensory data-collection, and the transferability of creativity. Of particular significance is the capacity for informed, selective pattern-breaking based on the ‘depth’ or ‘insider’ perspective of the domain expert; such ‘*expert variation and selective retention*’ provides creative choices and responses that are likely to be perceived by the field as creative: valuable, novel and surprising. The author is a renowned Australian studio bassist, jazz musician, and music educator who also plays field hockey for Australia at Masters level. His recently completed Ph.D. thesis, based on a performance and composition career spanning 46 years, takes the form of an analytical autoethnography drawn from personal field notes, diaries and interviews as well as published record albums.

## Introduction

Major innovations often come from individuals who are new to a field, having acquired their expertise in an unrelated field (…), however, (…) it is only apparent in retrospect when such a cross-domain application of knowledge will actually prove fruitful (…) the application of extradomain knowledge must still undergo selection at both the individual and sociocultural levels.

(Simonton, 2015)

Following Simonton’s reminder that it is the ‘field’ (Csikszentmihalyi, in Sternberg, 1988, p. 325–339) that selects-as-worthy creative works, this paper addresses cross-domain application of knowledge (or inter-disciplinary transfer of creativities), and specifically, the ‘*domain-specific or domain-general nature of creativity’* debate brought into focus by the extensive research of Baer (1993, 1994a, 1998), Baer and Kaufman (2005) particularly his ‘Domain Specificity and the Limits of Creativity Theory’ (Baer, 2012).

Regarding the difficulty in testing for general creativity (Gardner, 1993, p. 20; Baer, 1994b,c), and in establishing whether some form of measurable general ‘c’ creativity, similar to the general ‘g’ model of intelligence, actually exists (Baer, in Kaufman and Baer, 2012, p. 321), I offer some auto-ethnographic observations (and a few distinctions) from my own inter-disciplinary career experience. Having acquired the habitus and unique ‘insider perspective’ of both field hockey and jazz, a comparison of creative practice between the two followed naturally. The focus on jazz improvisation and field hockey is deliberate and pragmatic. These are two areas where I have a unique insider perspective, and where creativity is real-time, rather than multistage. For the purposes of this discussion, I treat.

Sawyer (2006, p. 115) posits that creative combinations often result when people switch fields, and that these multidisciplinary insights may be explained by *analogical thinking* (property mapping and structure mapping) – allowing the individual to perceive patterns that would not be apparent to someone working in only one domain. Following Johnson–Laird in Sternberg (1988), Baer (1993, p. 5) describes three kinds of creativity; real-time; multistage; and paradigm-shifting, and it is the first; real-time creativity, ‘under time constraints that make performance spontaneous, with no opportunity for revision’ that is the initial focus of this comparison between jazz and hockey creativities. As this research focuses specifically on real-time creativity, other passions where I have an ‘insider perspective,’ that is, songwriting and session/studio bass-playing, were not included as I consider them to be forms of multistage (rather than real-time) creativity. The inclusion of hockey in this discussion is not as unlikely as it may at first seem, as Weisberg acknowledges;

Athletic skills at the highest level also have creative components. In sports such as tennis, basketball, and hockey, to name a few, the basic activity is unstructured, and therefore requires constant improvisation (…) the results from studies of musical performers and athletes have relevance to the understanding of creative thinking.

(Weisberg, 1993, p. 235)

It is helpful to differentiate between what sports coaches describe as ‘closed,’ or ‘open’ skills (Poulton, 1957) with regard to relative creativity. ‘Closed skills’ are techniques that can be practiced without other-participant involvement (self-produced stimuli), and are usually non-creative; the serve in tennis, free throws in basketball, penalty-taking in hockey. ‘Open skills,’ by contrast, involves unstructured activity; reacting to other-participant moves, plays and positioning (external stimuli), providing multiple opportunities for creative thinking. It is in the notion of ‘open skills’ that improvisatory creativity resides; where the eyes, ears, brain and muscles respond to auditory (jazz) or visual (hockey) stimulus; visual, tactile and auditory stimuli are cognitively represented and evaluated; the central nervous system designs a response; and complex electrochemical signals are passed between the nervous system and on to endocrine and muscle systems (Pressing, 1988).

In the sporting arena, imaginative ‘plays,’ deceptive or ambiguous body-language signals, and surprising choices, are all part of the armory of high level athletes, especially for attacking players where invention and surprise are effective strategies for ‘unlocking’ well-organized defenses. Useful comparisons can be drawn between highly focused attention during ‘deliberate practice’ of ‘closed-skills’ on the hockey pitch, and when practicing the piano, double bass, or singing. Applying such closed skills in the field, ‘open-skill’ hockey matches with unpredictable opponents, conditions and variable situations, can similarly be compared with jazz improvisation, where immersion over decades provides generalities, patterns recognizable to the participants, likely outcomes, and a symbolic system of ‘triggers’ influencing behavioral responses.

…domains may overlap, either by having similar representations (i.e., some mapping function exists between the representations) or similar procedures. When this occurs, it is reasonable to expect that skill in one domain will correlate with skill in another.

(McShane, 1991, p. 318)

The skill correlation between jazz practice and improvisatory performance, and hockey practice and improvisatory performance is being highlighted here. The domains, and the specific skills or *tasks* required in both, are clearly dissimilar, however, recognizable patterns and similarities exist.

Baer (1993) focused attention on a *task-specific*, rather than a *domain-general* approach to testing and teaching for creativity; Sternberg (1985, p. 618) found that divergent thinking tests ‘capture, at best, only the most trivial aspects of creativity’, Plucker et al. (2004, p. 85) state ‘theorists over the past 25 years have moved toward more inclusive models of creativity in which divergent thinking plays an important but small role’. For Baer, while divergent thinking theory doesn’*t*-test well, it has been found to be somewhat successful for *teaching* creativity. Despite being contradicted by test results, Runco’s suggestion that ideational/divergent thinking, in the manner of Gardner’s (1983) ‘intelligences’ (Runco and Albert, 1990) is ‘certainly a step in the right direction’, according to Baer (1993, p. 92). To that end, Baer identifies an inviting research prospect to this auto-ethnographer, that being;

…the realm of matching specific skills to creative performance on specific tasks, a large research arena which I believe some of the most important future research in creativity will occur.

(Baer, 1993, p. 94)

This presents an intriguing possibility; if creativity is somehow transferable, perhaps it is through similarities in mapping functions or similar procedures; by aligning data-collection and discriminant pattern recognition across dissimilar domains. By observing multiple instances and possibilities for skill-matching across domains we may induce some useful generalizations informing the transferability of creativities.

## Discussion

The holy grail of creativity assessment research is a personality test to measure general creativity ability

(Sawyer, 2006, p. 58).

Performing improvised jazz at a professional level requires a form of musical creativity that is highly *domain-specific*; playing field hockey at an international level (similarly) requires a form of strategic, sporting creativity that is extremely *domain-specific*. However, while hockey skills may not be directly transferable to jazz improvisation, it is posited that certain broader, less specific creative skills may be. This does not suggest some form of *‘c’* or *domain-general* form of creativity, rather, it is posited that pattern-recognition and response may be seen as a form of transferable creativity. While the uninitiated may not see any connection between the two, a creative person with expert skills in both may connect the dots invisible to others; even beyond the educated perception of a person inculcated in only one domain as described by Schwandt;

“*What we seek is a heightened awareness or educated perception … that comes from intimate familiarity with the phenomenon”*

(Schwandt, 1994).

Such a dual-domain expert, inculcated with the necessary *habitus* Bourdieu (1983, p. 2), Bourdieu (1986, p. 170) in both realms, would have the capacity to make the type of unique, informed observations necessary to distinguish the remotely analogous connections between such dissimilar pursuits (Poincaré and Halsted, 1913; Mednick, 1962; Simonton, 2007; Simonton, in Sternberg and Davidson, 1995, p. 465–494; Weisberg, 1993, p. 93). Such a person would also have the capacity to see commonalities and recognizable patterns that non-experts would miss, to hypothesize circumstances in which these patterns may be generalized and applied elsewhere, and to theorize therefore, how such generalizations could be transferred as *domain-general* creative skills.

Just as jazz band-leaders appreciate and honor the responsive ensemble improviser on the bandstand, hockey coaches place a high value on the player who thinks tactically *on-the-run*, can execute the coach’s strategic plan, and improvise a way out of difficult pressure situations during the game. Both the improvising jazz musician and the strategic hockey player are collecting data (aural, and visual, respectively) as they interact and respond to the actions of others. For the creative practitioner in either domain, the capacity for discriminant *pattern recognition;* recognizing, identifying and responding to important stimuli from the immediate environment, either on the bandstand, or on the hockey pitch; is significant and highly valued.

In the metacognitive environment of the creative jazz scenario, the ability to recognize patterns embedded within harmonic routines, rhythm, melodic motifs, themes, chord progressions, and ‘licks’ is paramount. Without such ability, the musician (and receptive audience members) would be overwhelmed at the sheer density of musical data being presented at any given moment. As the players encode the performance with the blues, bebop, cool, and avant-garde traditions – the secret language and peculiar norms of the jazz idiom – the enlightened audience, who share a deep immersion and possess their own habitus in the jazz sub-culture, decodes the music, appreciates, and applauds the subtle expressions, quotes, citations, and the melodic and blues references interwoven into the performance.

Within the elite hockey culture, certain strategies, spatial positioning, skills, and approaches are deeply inculcated by players to improve their individual and team performance, and the introduction of an unfamiliar (novel or non-obvious) domain-specific skill well-executed (useful) is often very effective to creatively dismantle a well-drilled defense (as most state and national teams are). In creativity terms, if the striker’s visual and kinaesthetic movements are obvious and non-creative, they will appear to the defender as familiar, near-analogical references (Weisberg, 1993, p. 17, 20) that ‘converge’ on the defender’s experience, and the defender responses will be familiar, well-rehearsed, and likely to be successful. If the movements of the striker are, however, novel, usefully executed, and surprising (i.e., creative) the defender must respond to remote-analogical (Weisberg and Hass, 2007; Cunningham et al., 2009; Simonton, 2011) triggers that ‘diverge’ from the defender’s experience and do not automatically invoke defensive patterns. (Pressing, 1988) showed that reaction times with only *one chosen motor response* fall between 100 and160 ms (…) whereas reaction times to *unexpected* situations take up to 400 or 500 milliseconds. That 300 ms – might the difference between eliminating a defender (or not) and scoring (or not scoring), a goal. So is there some comparable capacity for pattern recognition that manifests itself in the jazz ensemble?

Parallels exist in the jazz improvisatory realm, where, based on my own experience working as a bassist with the cream of Australian jazz performers, a significant part of the function of a jazz bassist is to provide harmonic, rhythmic, and melodic support for the singer, soloist, and other accompanists on the jazz bandstand. Well-chosen creative responses to a soloist’s performance can provide the platform to propel an otherwise-unremarkable jazz solo into an engaging one. The ability to anticipate what those musicians expect, desire, or need to hear from the bass instrument at any given moment is what separates a great bassist from a poor one; well-rehearsed motor responses are much faster than significant voluntary compensations; the reaction times of the highly trained bassist to the “introduced novelties of improvising players” (Pressing, 1988), are much faster than those of the less-trained bassist. This use of pattern recognition (based upon *aural* data-collection) to inform musical choices allows the jazz bassist to please fellow musicians, singers and the audience. Sometimes it is *providing* patterns (bass ‘riffs’) that are pleasing because of their predictability. Sometimes it is *breaking* patterns for surprise, color, delaying the expectations of the audience, or creating tension and release. Sometimes it is simply being cognisant of the patterns, with no action taken.

Similarly, the use of pattern recognition (based upon *visual* data-collection) to inform sporting choices allows the elite hockey player to support fellow athletes, coaches and fans by executing patterns (off-the-ball actions) that are effective because of their strategic predictability. Sometimes it is *breaking* patterns for surprise, deception, confounding the expectations of the opposition, or creating goal-scoring opportunities. Sometimes it is simply being cognisant of the patterns, with no action taken (merely ‘holding’ a key opposition defender away from the anticipated play).

Whilst the task-specific activities involved in bass-playing and field hockey then are very different, and domain-specificity is clearly evident, the activities are near-analogous in that they both use pattern recognition (and pattern deception) to influence the audience (or opposition). What is generalizable and transferable across disciplines then is a capacity for data-collection and pattern-recognition, facilitated by expert habitus in both fields.

## Implications

It is unsurprising that the transfer of *domain-specific* creativity tends to be more prevalent in related domains than in unrelated ones. If one has achieved a high skill level through directed study in one domain, then many of the same skills will obviously facilitate creativity in the near-analogous situations of a related discipline. Dissimilar domains provide an opportunity to look at what can be transferred when task-specific skills have little in common. Due to this unique dual-domain perspective, when a person with expertise across two very different fields of endeavor does manage to transfer skills across unrelated disciplines, it is likely that the resultant creative products or propulsions will be different somehow. Following Sternberg et al.’s (2002) *Propulsion Theory of Creativity,”* the type of Advanced Forward Incrementation, Synthesis, or other propulsions that *reject or challenge* ‘where we are now’ come into play, rather than the more common Forward Incrementation and other creative propulsions that *accept* ‘where we are now’. Skills possessed across unrelated disciplines precipitate more divergent, remote-analogical solutions than those convergent solutions implied by closely related, domain-specific transfers.

However, without a willingness and preparedness to use divergent thinking methods like blind variation and selective retention (BVSR) including trial and error or random experimentation and a degree of risk-taking (Campbell, 1960; Simonton, 2011), thinking will tend to be convergent and propulsions will tend to be of the forward incrementation variety; resulting in creativity unlikely to be considered by the field as paradigm shifting. The hockey player becomes predictable, the jazz improviser’s playing somewhat stale, and the songwriter’s artifacts popular, but unremarkable. It is posited that a highly skilled, deeply immersed dual-domain expert might apply their unique multidisciplinary habitus in a form of ‘expert’ variation and selective retention (EVSR) where experience allows a form of discriminant pattern recognition unavailable to lesser songwriters. From this informed EVSR perspective, trial and error, risk-taking and solution-seeking is much more likely to be successful than the random choices of a novice whose variations are ‘blind’; successful creative outcomes are not simply guess-work, they are influenced by expert habitus, ‘intuitive feelings of warmth’, and substantial prior trial and error.

## Conclusion

As Simonton suggests, persons with expert skills in at least two areas are simply more likely to make the unique distinctions that less expert, less inter-disciplined persons cannot, and thereby discover what is transferable. Returning to the question posed in the introduction; ‘*What skills or creativities are transferable across dissimilar domains’*, it is posited that on both the bebop bandstand and the hockey field, data collection and discriminant pattern-recognition are significant. Based on aural or visual input stimuli, the capacity to recognize and support, match, complement, or break patterns, responding in the context of the encoded habitus of the domain to desirable effect, represents a transferable creativity. It is possible that this type of creativity transfer may have wider applications, that is, beyond merely jazz improvisation and team sports, where, for example, the capacity to collect visual data and use discriminant pattern-recognition in the field of architecture may be used in the auditory data realm of sound mixing in the recording studio. Or, during a motor race, a highly skilled driver may use an acutely developed sense of auditory pattern recognition to identify nuances of race-car performance (based on the sound of the engine or tires) to inform, confirm or refute the pit-crew’s analysis of visual computer data. Alternately, that same race-driver may ‘creatively’ overtake another driver (having observed their driving patterns and invented a non-obvious overtaking sequence that is difficult to ‘defend’). While a world-champion race driver or an expert sound-mixer (with substantial commercial capital at stake) might be reluctant to disclose (under interview) their capacity to transfer creativity in such a way, such disclosure (if given) may provide further evidence in support of this perspective. Furthermore, the capacity to generalize and subsequently transfer *domain-general* skills across domains, the realm of the field shifter or multidisciplinary expert, may be one factor that stimulates the creative person to explore propulsions that reject or challenge the current paradigm, by introducing fruitfully asynchronous, remotely analogous ideas that (if accepted by the field as worthy) are seen to be highly creative.

## Author Contributions

The author confirms being the sole contributor of this work and approved it for publication.

## Conflict of Interest Statement

The author declares that the research was conducted in the absence of any commercial or financial relationships that could be construed as a potential conflict of interest.
